# Mixed-Methods Analysis of Emotional Quality in Sports Organizations: Facial Expressions of Child Users of Sports Services as Data

**DOI:** 10.3389/fpsyg.2020.01199

**Published:** 2020-07-10

**Authors:** Verónica Morales-Sánchez, Rocío Pérez-López, Rafael E. Reigal, Antonio Hernández-Mendo

**Affiliations:** ^1^Department of Social Psychology, Social Anthropology Social Work and Social Services, University of Malaga, Málaga, Spain; ^2^Junta de Andalucía, Seville, Spain; ^3^Faculty of Medicine, University of Malaga, Málaga, Spain

**Keywords:** Mixed Methods, emotional quality, sports organizations, facial expressions, children

## Abstract

Proper quality management of sports services requires knowledge of the needs, perceptions, expectations, and emotions of users. Facial expressions provide relevant information about the immediate perception of stimuli. The objective of this study was therefore to analyze how a group of child users of sports services expressed their satisfaction with the programs in which they participated through facial expressions during an in-depth interview tailored to their age. Nineteen child users of a sports organization (aged from 5 to 12 years) were interviewed, involving both qualitative and quantitative analyses. The analysis of facial expressions was performed using Face Reader v.6.1.10, which automatically analyzes eight categories of facial expressions (six basic emotions, one neutral, and one unidentified). The data collection and transformation procedures fully satisfied the requirement for flexibility and provide new perspectives for incorporating data into the study of biopsychosocial factors in everyday contexts using a Mixed-Methods approach. In addition, the generalizability analysis itself, carried out to determine the levels of reliability, generalizability and precision of the sample (with relative and absolute G indexes of between 0.86 and 0.93 for the three analyses performed), was also a Mixed-Methods procedure.

## Introduction

Users of a sports service focus not only on how useful it is to them; an especially important issue is how it is associated with the experience of an emotion (Fernando-García et al., 2020). Hence the importance of evaluating the elements related to the pleasure experienced by the user, a factor that is becoming increasingly important in assessing the quality of a service (Pérez-López et al., 2015). In this sense, emotions play a very important role in sports practice and are emerging as a key facet of user satisfaction in sports centers (Silla-Merchán et al., 2014; Pedragosa et al., 2015).

There are studies suggesting that in the practice of directed sports activities emotional experience is a factor that can contribute to satisfaction with the activity practiced and is more likely to promote future practice intentions (Lee et al., 2016). Satisfaction with the performance of a sporting activity, therefore, is not only a cognitive phenomenon but also includes other determinants, such as affective states (Wirtz and Bateson, 1999; Wirtz et al., 2000; Martínez-Tur et al., 2001). Ries and Sevillano (2011) state that positive emotions will encourage the intention to practice sport and create a greater willingness to increase the frequency and duration of that activity, whereas the opposite occurs with negative emotions. Therefore, in the context of a sports organization, knowing how emotional experiences influence users and how they relate to satisfaction and the perceived quality of services is a vital issue that has already been highlighted by several authors (Bigné and Andreu, 2004; Jiang and Wang, 2006; Ladhari, 2009; Ozgen and Duman Kurt, 2012).

It is especially important to include the study of emotions in the user satisfaction process, because most services are based on the experience of the users of a given service (Liljander and Strandvik, 1997; Foxall and Greenley, 1999; Benkenstein et al., 2003; Lewinski et al., 2016). For this reason, in conceptualizing and measuring the quality of the service provided it is considered important to differentiate between a hedonistic and a utilitarian type of service. In the evaluation of a utilitarian service, rational factors will have the greatest weight, while in the other type, satisfaction with the service will be determined largely by emotional responses (Jiang and Wang, 2006). From the hedonistic perspective, the perception of quality will be associated with the presence of pleasant or agreeable properties intrinsic to the service being evaluated (Mano and Oliver, 1993). Following this line of argument, an increasing number of authors have raised the need to take account of the more emotional factors in concepts of quality of service (Price et al., 1995; Gwinner et al., 1998; Peiró et al., 2005; Potocnik et al., 2010).

To improve the emotional experience of users in organizations, several issues need to be considered. For example, interaction between workers and users will be of vital importance, and good communication between them should be encouraged if the experience of positive emotions is to be increased (Ozgen and Duman Kurt, 2012; Romani et al., 2012). Similarly, Molina-García et al. (2016) argue that it is essential to create attractive activities and pleasant environments to foster a more positive emotional experience and contribute to increasing user satisfaction in sports organizations.

In this study we used the Three-Dimensional Model of Service Quality (Sánchez-Hernández et al., 2009). This model posits three facets of quality. (1) Functional quality of service refers to the efficiency with which the core service is provided. This dimension includes factors such as reliability, safety, and responsiveness. (2) Relational or emotional service quality focuses on the emotional factors (extras, genuine understanding, and empathy) related to interaction during the service that go beyond the core service and demonstrate affection and esteem toward the user. Finally, (3) tangible factors refer to opinions of the more physical or material elements that accompany the service, such as the facilities or equipment used.

It is possible to ascertain the emotions of users by analyzing their facial expressions, a subject that has been studied for more than a century (Darwin, 1872/1998). This type of analysis has been a fundamental guiding principle for the main theories of emotion (Tomkins, 1962; Izard, 1977; Izard et al., 1990; Ekman, 1992). Emotions can be caused by many things, such as an unexpected situation, remembering or talking about a past experience, or seeing the emotional reactions of another person (Yu and Ko, 2017). Of all the emotions reflected in facial expressions, six have been considered innate. These are known as the basic emotions: joy, sadness, fear, disgust, surprise, and anger (Ekman, 1993, 2003).

The work of Cameron et al. (2018), though conducted with robots, discusses the influence of facial expressions according to gender and its role in social relations. The importance of gender in facial expressions has also been confirmed by Bachmann et al. (2019), using FaceReader and a psychiatric population. Terzis et al. (2013), estimating that FaceReader is capable of measuring emotions with an effectiveness greater than 87% during a CBA (Cognitive Behavioral Assessment), point out that analysis by gender highlights the differences between the spontaneous emotions of each gender.

There are various systems for measuring facial expressions, but the most widespread and commonly used is the Facial Action Coding System (FACS) (Ekman and Friesen, 1976). This system assigns a number to each facial configuration, produced by movements of a set of muscles, and these numbered individual configurations are called action units (AU) (Ekman, 2003). It also uses EMFACS, an acronym for “Emotion FACS,” based on using the FACS to detect emotions through facial expression. Both systems use hand coding.

This procedure certainly attributes an important role to facial expression in emotional response, as reflected in the facial feedback hypothesis (Tomkins, 1979), which postulates that the activity of the facial muscles is mainly responsible for emotional experience through a muscular sensory feedback process, according to which facial expressions can evoke emotional reactions in the person making them. This was a starting point for the systematic study of facial expression, giving functional value to the different facial muscles that could be used as units of action to classify different emotions (Ekman, 2003).

Significant progress has been made in automated analysis of facial expression. Over the past few decades, tools have been developed that overcome the limitations of FACS coding. The rapid development of information technology has enabled these analyses to be carried out automatically, while achieving greater validity, speed, and accessibility (Cootes et al., 2001; Viola and Jones, 2001; Beumer et al., 2006; Valstar et al., 2011; Swinton and El Kaliouby, 2012; Lewinski et al., 2014).

FaceReader is one of the tools used for the analysis of facial expression of emotions (van Kuilenburg et al., 2005; Lewinski et al., 2014), although there are others, such as AFFDEX and FACET from iMotions (Stöckli et al., 2018). Recent studies have indicated that this software is an effective tool for analyzing emotions, with 90% accuracy (Loijens and Krips, 2013). A similar degree of accuracy is achieved in the work of Terzis et al. (2013) and Lewinski et al. (2014). FaceReader is an instrument that allows automated direct observation of facial expressions. This process can, in itself, be considered a Mixed Method (Anguera and Hernández-Mendo, 2016). This study also uses a CONNECT strategy of integrating qualitative and quantitative elements.

Mixed Methods have been defined by classical authors as “the type of research in which a researcher or team of researchers combines elements of qualitative and quantitative research approaches (e.g., use of qualitative and quantitative viewpoints, data collection, analysis, and inference techniques) for the broad purposes of breadth and depth of understanding and corroboration” (Johnson et al., 2007, p. 123). Mixed Methods are used extensively in educational, psychological, sports, health, and social contexts. They do not constitute a unitary whole; the approaches are plural, but they converge in the essential point: the term describes “a study that mixes or combines quantitative and qualitative methods, techniques, concepts, or language into a single study or series of linked studies,” as Fakis et al.(2014, p. 139) define it. This core, however, is remarkably broad, which is consistent with the extensive and intensive deployment of the discipline (Anguera and Hernández-Mendo, 2015). The complementarity that Mixed Methods propose can be implemented from a range of positions, guided by the designs included in the scientific literature of recent years (Johnson and Onwuegbuzie, 2004; Teddlie and Tashakkori, 2006, 2009; Creswell and Plano Clark, 2007; Greene, 2007; Leech and Onwuegbuzie, 2009; Morse and Niehaus, 2009; López-Fernández and Molina-Azorín, 2011; Anguera et al., 2012, 2014; Guest, 2012) that have made it possible to establish three strategies for integrating qualitative and quantitative elements: MERGE, CONNECT, and EMBED.

The objective of this study was to evaluate the satisfaction of a group of children with the sports programs in which they participate by analyzing their facial expressions during an interview conducted for this purpose.

## Materials and Methods

### Design

We designed a systematic direct observational study (Anguera et al., 2017) to analyze in-depth interviews with children in a sports context. The Mixed-Methods approach involves the collection, analysis, and interpretation of qualitative and quantitative data for the same purpose and within the same study. Systematic observation of child users in a sports organization was carried out as a system integrating quantitative and qualitative analysis and satisfying both the rigorous standards of scientific research and the flexibility required to study the unstructured ecological context of a sports organization (Plano Clark et al., 2015; Portell et al., 2015; Anguera and Hernández-Mendo, 2016). Integrating quantitative and qualitative data can dramatically increase the value of research with Mixed Methods (Fetters et al., 2013).

In this case, the methodological approach must be extremely rigorous, since these are situations in which the substantive areas merge with the multiple realities of everyday life. Considering the nature and requirements of the study, they justified the use of a Nomothetic/Isolated/One-dimensional observational design, corresponding to quadrant III of the designs in observational methodology (Anguera et al., 2001, 2011; Sánchez-Algarra and Anguera, 2013). The design was (a) *nomothetic*, because a plurality of children were observed, acting individually; (b) *isolated*, because data were collected during a session for each child, but each session was recorded in its entirety, without interruption (intra-sessional monitoring); and (c) *one-dimensional*, because the instrument of observation had only one dimension (the eight categories established by FaceReader).

### Participants

A sample of 19 (*n* = 19) participants was obtained, of whom 8 were boys (42.10%) and 11 were girls (57.89%), aged between 5 and 12. They all participated in sports activities in Alcalá la Real (Jaén, Spain): swimming, hockey, football, acrobatic gymnastics, tennis, basketball, and rhythmic gymnastics. The sampling was intentional, seeking to achieve stratification with proportional allocation.

Parents were informed in advance of the recording of the interviews and asked for their signed consent, in accordance with the Helsinki Declaration (World Medical Association, 2013). Approval was also obtained from the Ethics Committee of the University of Málaga (No. 243; CEUMA Registry: 18-2015-H).

### Instruments

The individual interviews were conducted with questions about the sports activities, the instructor, the equipment, the sports facility/space, and the organization. The questions were formulated following the Three-Dimensional Model of Service Quality (Price et al., 1995; Sánchez-Hernández et al., 2009).

The individual interviews were recorded with a Sony HDR-CX505VE digital camera. Facial recognition of emotions was performed using FaceReader software (Noldus, 2015), version 6.1.10. FaceReader is a program for analyzing facial expression. It can detect emotional expressions of the face, identifying eight configurations, six related to basic emotions (happy, sad, angry, surprised, scared, and disgust), a neutral and one that does not identify the six emotions indicated above (unidentified). It also detects movements of the face (right and left, opening and closing the eyes, opening and closing the mouth, raising and lowering the eyebrows). It recognizes a face and then models it in three dimensions and finally analyzes it to classify the emotions that the person expresses.

The software used, in addition to FaceReader, was the SDIS-GSQ program (Bakeman and Quera, 2011) for sequential analysis, the Hoisan coding and analysis program (Hernández-Mendo et al., 2012) to carry out the analysis of polar coordinates, the SAS statistical program v.9.1 (Schlotzhauer and Littell, 1997; SAS Institute Incorporated, 1999) to analyze variance components, the SAGT program (Hernández-Mendo et al., 2016) to perform generalizability analyses, and the Theme program (Pattern Vision Ltd and Noldus Information Technology, 2004) for calculating T- patterns.

### Procedure

Individual interviews were conducted with each of the children separately. To capture the face properly, the video camera was placed on the tripod, so that it could focus on the children’s faces. Natural lighting was used and in no case was there any additional lighting.

The duration of each individual interview was adjusted to each child’s attention span and ability to concentrate, allowing for a detailed description of their experiences as a participant in sports activities.

Once the facial expressions had been coded using FaceReader software, the data were transformed into code matrices containing purely qualitative information (Anguera et al., in press). This transformation is achieved by organizing the dominant emotion into a single column ready for quantitative analysis.

## Results

First, a variance components analysis was performed for estimation of normality. We decided to perform a least-squares variance analysis (VARCOM Type I) and a maximum likelihood (GLM) components analysis and check whether the error variance was the same in the two procedures. A three-faceted model [y = s| p| e] was used, where s = sex, p = person, and e = emotion. The error variance with the two procedures proved to be equal (GLM error = 34159.14021/VARCOMP error = 34159 for the model without interactions, and zero for the model with interactions). With these estimated results showing the equality of error variance in both a least-squares and a maximum-likelihood procedure, it can be assumed that the sample is linear, normal, and homoscedastic (Hemmerle and Hartley, 1973; Searle et al., 1992).

Subsequently, a generalizability analysis with a crossed design was performed on the [s][e]/[p] model and it was shown that the relative *G* index (reliability) = 0.95 and the absolute *G* index (generalizability) = 0.94 (see Table 1) (Blanco-Villaseñor et al., 2014).

**TABLE 1:** Models used in generalizability analysis.

Name of the values	Model [s] [e]/[p]	Model [e]/[p] for girls	Model [e]/[p] for boys
*s*	(2; INF)		
*p*	(19; INF)	(11; INF)	(8; INF)
*e*	(8; INF)	(8; INF)	(8; INF)
Total number of observations	304	88	64
Relative *G* coefficient	0.957	0.929	0.876
Absolute *G* coefficient	0.946	0.908	0.855
Relative error	6.151	18.031	39.711
Absolute error	7.737	23.815	47.718
Standard deviation of relative error	2.480	4.246	6.302
Standard deviation of absolute error	2.781	4.880	6.908

Another generalizability analysis with a crossed design was then performed, first by gender, with model [e]/[p], where e = emotion and p = participants. For girls, the relative G (reliability) = 0.92 and absolute G (generalizability) = 0.90. In boys, values of relative G coefficient (reliability) = 0.87 and absolute G (generalizability) = 0.85 were obtained (see Table 1) (Blanco-Villaseñor et al., 2014).

Subsequently, two types of analysis were performed based on the FaceReader results, T-patterns, and polar coordinate analysis.

### T-Pattern Analysis

T-pattern analysis is an analytic technique in the field of observational methodology that makes it possible to detect T-patterns (regular behavioral structures) using THEME software, which analyzes observational data in search of critical interval relationships in an upward direction, from a simple T-pattern (two events that are related at a first level) to complex T-patterns (comprising a number of events related at various levels) (Magnusson, 1996, 2000, 2018).

The assumption underlying the T-pattern detection method is that complex human behaviors have a temporal structure that cannot be fully detected through traditional observational methods or mere quantitative statistical logic (Magnusson, 1996, 2000, 2018). By detecting T-patterns, or time patterns, this method can detect structural analogies across many different levels of organization and allow a major shift to be made from quantitative to structural analysis.

T-pattern detection studies have been carried out in many different scientific fields and also in the field of sport (Borrie et al., 2002; Chaverri et al., 2010; Gutiérrez-Santiago et al., 2013; Lapresa et al., 2013a, b, 2015; Zurloni et al., 2014; Tarragó et al., 2015; Aragón et al., 2016). Furthermore, T-pattern detection has wide applicability in many fields, such as the study of emotions (Merten and Schwab, 2005) and education (Castañer et al., 2013; Suárez et al., 2018), etc.

Since human behavior observation records have a temporal and sequential structure, an analytic tool that can describe this structure can only enhance understanding of the target behavior(s). For instance, the discovery of hidden T-patterns could help coaches to better predict the behavior of competitors through an integrated system allowing greater depth of analysis.

The calculations of the methods used in this study were made with the THEME v.6 Edu (T-pattern detection) program (Magnusson, 1996, 2000, 2018), which is available free of charge.

### Analysis of Polar Coordinates

Polar coordinate analysis was proposed by Sackett (1980) and optimized through the “genuine retrospectivity” technique of Anguera (1997), which improves on Sackett’s original approach. This technique makes it possible to achieve a drastic reduction of data and a vectorial representation of behavior in the interrelations between the different categories that make up the proposed taxonomic system (Hernández-Mendo and Anguera, 1999; Gorospe and Anguera, 2000).

The technique is based on a sequential analysis of the prospective (Sackett, 1980) and retrospective delays in the successive behaviors that occur, using the genuine technique (Anguera, 1997). The values derived from the calculation of the conditioned probability will make it possible to obtain the Zsum parameter:

$$\text{Zsum}=\frac{\Sigma \,z}{\sqrt{\text{n}}};\bar{\text{x}}=0;s\,x=1$$

where *n* is the number of delays (Cochran, 1954). The distribution of this Zsum parameter has a mean equal to zero and a standard deviation equal to one. By obtaining these values, we establish the interrelational behavior map or polar coordinate map (Gorospe and Anguera, 2000). In order to produce the behavioral maps, the value of the vectors needs to be determined (they must be equal to or greater than 1.96 to be considered significant). The modulus or length of the radius is obtained from the square root of the sum of the square of the Zsum of the *X* (forward) and the square of the Zsum of the *Y* (backward):

$$R\,a\,d\,i\,u\,s=\sqrt{x^{2}+y^{2}}$$

The angle of the vector (φ) (which will depend on the quadrant in which it is found) marks the nature of the relationship (Castellano and Hernández-Mendo, 2003). This angle (φ) is calculated as:

$$\phi =\frac{\mathrm{Arc}\,\mathrm{sine}\,\mathrm{of}\,Y}{\text{Radius}}$$

Polar coordinate analysis also has wide applicability in several fields of knowledge, such as clinical psychology (Arias-Pujol and Anguera, 2017; Rodríguez-Medina et al., 2018), sports (López-López et al., 2015; Morillo-Baro et al., 2015; Morillo et al., 2017; Menescardi et al., 2019), environmental psychology (Pérez-Tejera et al., 2018), etc.

#### Girls

**FIGURE 1:**
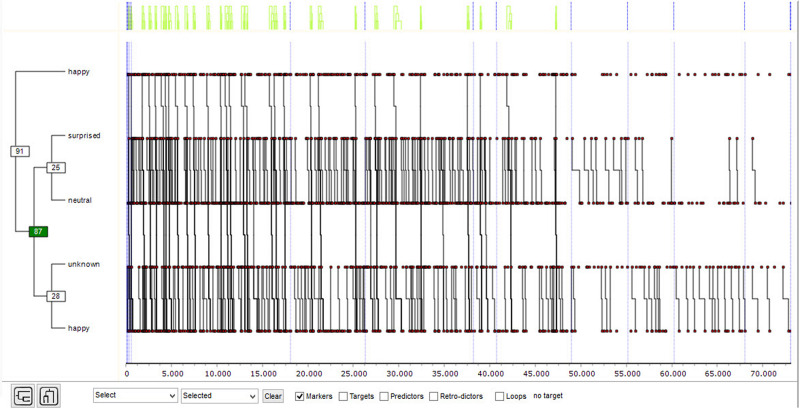
T-pattern 1 for girls (significance level = 0.005; minimum number of occurrences = 30).

The girls’ records were analyzed using the THEME program to detect T-patterns that show the structure of emotional transitions. The T-pattern 1 obtained (Figure 1) has the following structure: {happy [(surprised neutral)(unknown happy)]}.

There is a clear recurrence between *surprised* and *neutral*, as well as between *unknown* and *happy*.

In order to have evidence of both recurrences, polar coordinate analysis was applied, carried out on a transitory basis from the two existing possibilities.

##### Surprised/neutral relationship

On the one hand, *surprised* was considered as a focal behavior and *neutral* as conditioned, making them activate each other, since the *neutral* vector is in Quadrant I. And on the other hand, *neutral* was considered as a focal behavior and *surprised* as conditioned, also leading to their mutual activation, since the *surprised* vector is in Quadrant I (Table 2).

**TABLE 2:** Polar coordinate analysis focusing on *surprised*/*neutral* and *neutral*/*surprised* relationships, corresponding to the girls’ records.

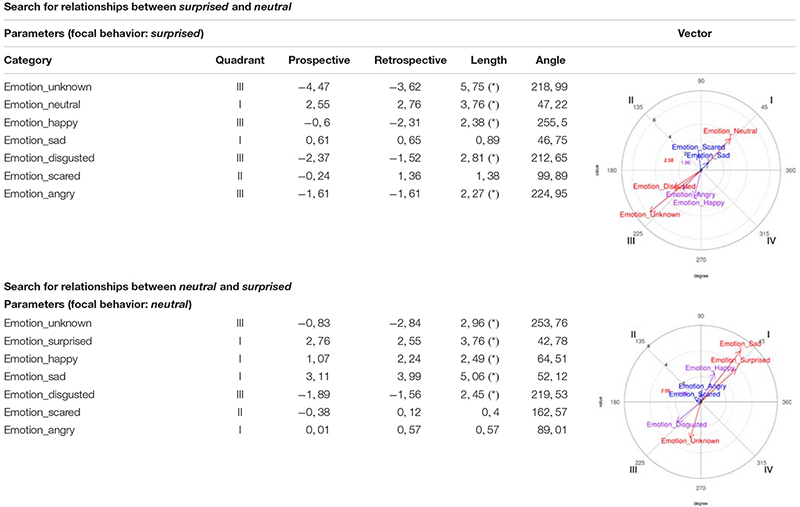

*The meaning of ^∗^ is a significant vector (z ≥ 1.96, p ≤ 0.05).*

##### Unknown/happy relationship

On the one hand, *unknown* was considered as a focal behavior and *happy* as conditioned, making them activate each other, since the *happy* vector is in Quadrant I. And on the other hand, *happy* was considered as a focal behavior and *unknown* as a conditioned behavior, also causing them to activate each other, since the *unknown* vector is in Quadrant I (Table 3).

**TABLE 3:** Polar coordinate analysis centered on *unknown*/*happy* and *happy*/*unknown*, corresponding to the girls’ records.

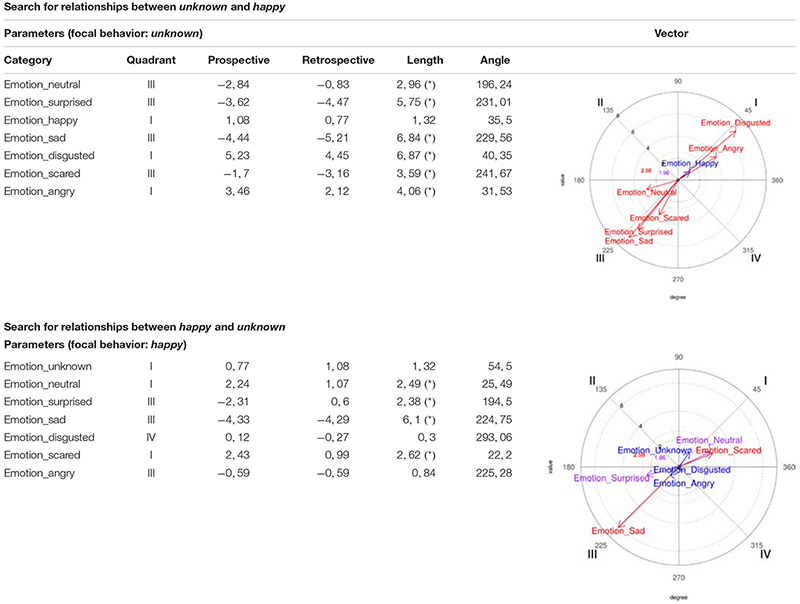

*The meaning of ^∗^ is a significant vector (z ≥ 1.96, p ≤ 0.05).*

As indicated above, the vectors of Quadrant I show the existence of reciprocal activation relationships between the focal behavior and the corresponding conditioned behavior.

#### Boys

**FIGURE 2:**
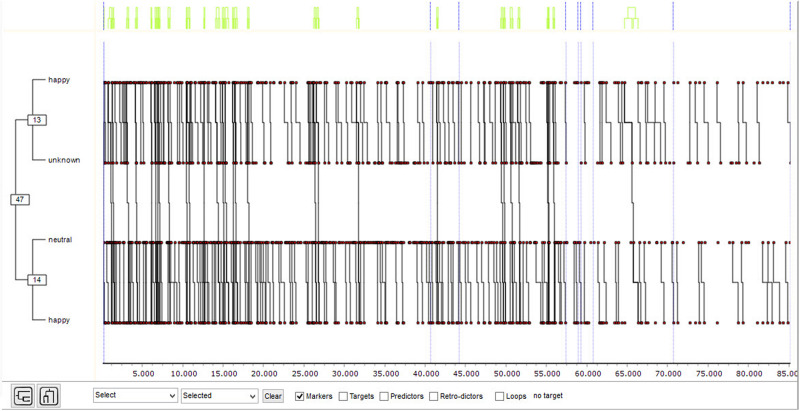
T-pattern 1 for boys (significance level = 0.005; minimum number of occurrences = 30).

The boys’ records were analyzed with the THEME program to detect T-patterns that show the structure of emotional transitions. The T1 pattern obtained (Figure 2) has the following structure: [(happy unknown)(happy neutral)].

There is a clear recurrence between *happy* and *unknown* expressions, as well as between *neutral* and *happy*.

In order to have evidence of both recurrences, polar coordinate analysis was applied on a transitory basis from the two existing possibilities.

##### Happy/unknown relationship

On the one hand, *happy* was considered as focal behavior and *unknown* as conditioned, making them activate each other, since the *unknown* vector is in Quadrant I. And on the other hand, *unknown* was considered as focal behavior and *happy* as conditioned behavior, again leading them to activate each other, since the *happy* vector is in Quadrant I (Table 4).

**TABLE 4:** Polar coordinate analysis centered on happy/unknown and unknown/happy, corresponding to the boys’ records.

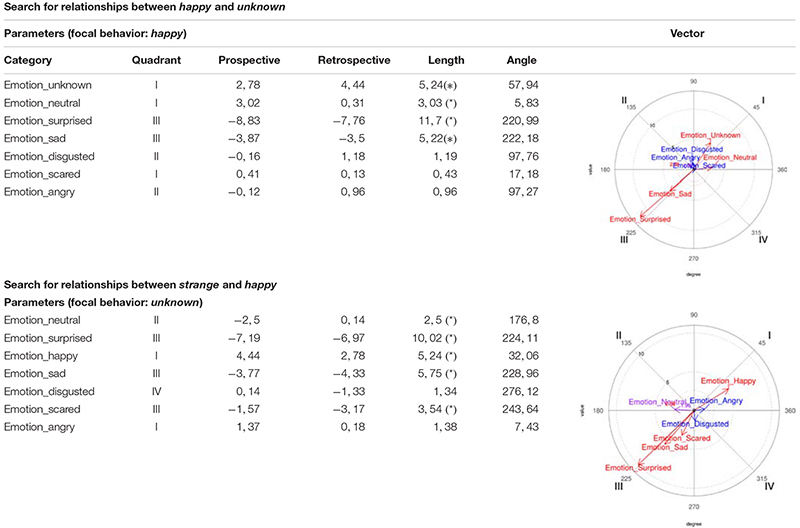

*The meaning of ^∗^ is a significant vector (z ≥ 1.96, p ≤ 0.05).*

##### Neutral/happy relationship

On the one hand, *neutral* was considered as focal behavior and *happy* as conditioned, making them activate each other, since the *happy* vector is in Quadrant I. And on the other hand, *happy* was considered as focal behavior and *neutral* as conditioned behavior, also leading them to activate each other, since the *neutral* vector is in Quadrant I (Table 5).

**TABLE 5:** Polar coordinate analysis centered on *neutral*/*happy* and on *happy*/*neutral*, corresponding to the boys’ records.

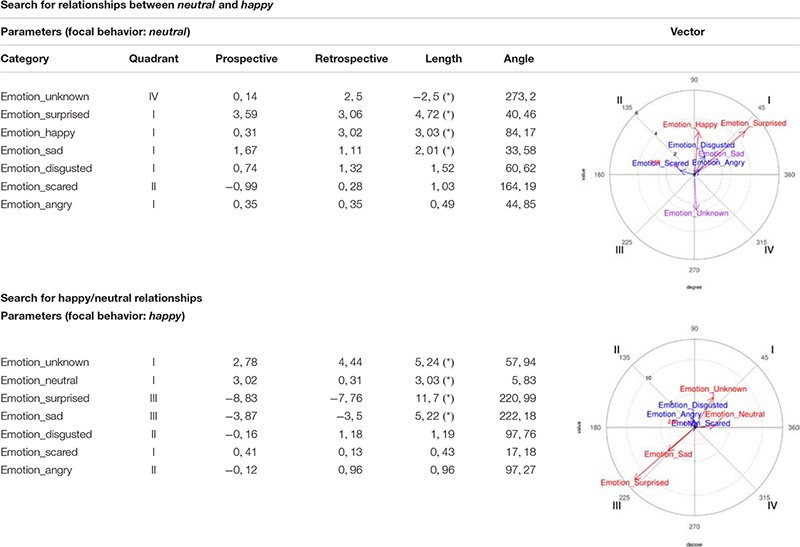

*The meaning of ^∗^ is a significant vector (z ≥ 1.96, p ≤ 0.05).*

## Discussion

The aim of this study was to assess satisfaction with sports programs in which a group of children participated. For this purpose, interviews were recorded and facial expressions were analyzed. This is in line with previous work that has highlighted the importance, from a hedonistic perspective, of analyzing emotional factors in order to assess the quality of the services provided to users and their satisfaction with them (Peiró et al., 2005; Jiang and Wang, 2006; Potocnik et al., 2010).

The study integrates the data from interviews focusing on facial expressions, recorded sequentially using FaceReader, with quantitative data analysis techniques, such as T-pattern detection and polar coordinate analysis and is thus recognizable as a Mixed-Methods approach. Generalizability analyses were also performed to establish the reliability and generalizability of the data collected. The results obtained were satisfactory and indicate not only that the sample is adequate in terms of reliability, but also that the results can be generalized, in relation both to the total sample and to the sample by gender.

In general terms, this study has detected recurrences that serve as a basis for checking the relationships between the respective codes of emotional states by polar coordinate analysis, with the dual intention of assessing the relationship between the two analytic techniques and establishing the differential profiles of boy and girl users. The results are relevant for several reasons.

First, there is an important correspondence between the results of the T-pattern analysis and those of the polar coordinate analysis. The data show considerable congruence between the dyadic relationships detected in the T-pattern analysis and the analysis of polar coordinates, as suggested by the results of previous studies (Castañer et al., 2017; Santoyo et al., 2017; Tarragó et al., 2017; Escolano-Pérez et al., 2019; Portell et al., 2019).

Second, the profiles found in boys and girls have some convergent features and others that suggest gender differences. In the T-pattern for girls, a clear relationship can be observed between the emotional state of *surprise* and *neutrality*. The analysis of the respective polar coordinates was performed taking the first element of each dyad (*surprised* and *unknown*) as focal and the second element (*neutral* and *happy*) as conditioned behavior in Quadrant I, which involves mutual activation. There is also a clear relationship between *unknown* and *happy* emotional states. The relevant polar coordinate analysis was performed considering the first element of each dyad (*unknown* and *happy*) as focal and the second (*unknown* and *happy*) as conditioned behavior in Quadrant I, again involving mutual activation (Codina et al., 2016).

The T-pattern for boys shows a clear relationship between *happy* and *unknown* emotional states. The relevant polar coordinate analysis was performed taking the first element of each dyad (*happy and unknown*) as focal and the second element (*unknown* and *happy*) as conditioned behavior in Quadrant I, which involves mutual activation. There is also a clear relationship between *neutral* and *happy* emotional states. The relevant polar coordinate analysis was performed considering the first element of each dyad (*neutral* and *happy*) as focal and the second element (*happy* and *neutral*) as conditioned behavior in Quadrant I, again involving mutual activation.

As can be seen, the emotional states prioritized by girls and boys partially coincide. However, they do show some peculiarities that characterize each gender and suggest differentiated emotional experiences. This phenomenon has already been highlighted in previous studies, which is consistent with the results obtained in this research (Terzis et al., 2013; Bachmann et al., 2019). The close relationship between the *happy* and *unknown* emotions is significant in both groups, and we consider that the contextual situation in the in-depth interview could explain the specific nature of the emotions related to sports practice.

One of the recurring expressions throughout the analysis is “happy,” in both girls and boys. This fact is consistent with the research conducted in relation to the Three-Dimensional Model of Service Quality (Sánchez-Hernández et al., 2009), which is concerned with emotional service quality focused on interaction during service and which shows affection and esteem for the user.

The limitations of this study lie in the sampling. It would be valuable to sample over different cities and to increase the age range. In relation to Mixed-Methods strategies, the use of a MERGE strategy in addition to a CONNECT strategy, with content analysis, would give the study greater power and flexibility.

## Data Availability Statement

The datasets generated for this study are available on request to the corresponding author.

## Ethics Statement

The studies involving human participants were reviewed and approved by 18-2015-H (Ethics Committee of the University of Malaga). Written informed consent to participate in this study was provided by the participants’ legal guardian/next of kin.

## Author Contributions

All authors listed have made a substantial, direct and intellectual contribution to the work, and approved it for publication.

## Conflict of Interest

The authors declare that the research was conducted in the absence of any commercial or financial relationships that could be construed as a potential conflict of interest.
